# Valacyclovir-associated acute kidney injury and encephalopathy in an elderly woman with normal kidney function: a case report

**DOI:** 10.1007/s13730-022-00748-5

**Published:** 2022-11-18

**Authors:** Kazufumi Kato, Reiichi Murakami, Hiroshi Shiroto, Daiki Nagawa, Ikuyo Narita-Kinjo, Takeshi Fujita, Michiko Shimada, Hirofumi Tomita

**Affiliations:** 1 Department of Cardiology and Nephrology, Hirosaki University Graduate School of Medicine, Hirosaki, Aomori Japan; 2 Department of Internal Medicine, Hirosaki Stroke and Rehabilitation Center, Hirosaki, Aomori Japan; 3 Community Medicine, Hirosaki University Graduate School of Medicine, Hirosaki, Aomori Japan

**Keywords:** Valacyclovir, Acyclovir, Acute kidney injury, Encephalopathy

## Abstract

A 72-year-old Japanese woman was treated by 3000 mg/day of valacyclovir for the herpes zoster in her left back. She had been treated as hypertension with no renal insufficiency. In two days, she visited an emergency room of a regional stroke care center with dysarthria, dexterity disorder and gait disturbance. Neither head CT nor MRI found intracranial lesions, then, laboratory tests revealed that her serum creatinine level was 4.63 mg/dL. She was transferred and admitted to our hospital on the following day and received hemodialysis under the diagnosis of AKI due to acyclovir accompanied with encephalopathy. Afterward, her serum concentration of acyclovir revealed as 44 μg/mL, which is extremely high. Her neurological symptom disappeared in parallel with the decrease of serum concentration of acyclovir. She received 3 sessions of hemodialysis and discharged on the 8th day of admission with almost normal renal function and no neurological sequela.

**Supplementary Information:**

The online version contains supplementary material available at 10.1007/s13730-022-00748-5.

## Introduction

Valacyclovir, which is an oral acyclovir prodrug and has better bioavailability and longer plasma half time than acyclovir [1], is an antiviral agent which is widely used for the patients with herpetic infections. It has been previously reported that the nephrotoxicity of acyclovir often causes acute kidney injury (AKI) [2, 3]. Also, both acyclovir and valacyclovir sometimes cause drug-associated encephalopathy by the delay of drug metabolism mainly in patients with renal insufficiency [4, 5]. In this report, we present a case of healthy elderly woman with normal kidney function who suffered from AKI accompanied with encephalopathy by the oral administration of regular dose of valacyclovir.

## Case report

A 72-year-old Japanese woman started to take 3000 mg/day of valacyclovir for the treatment of herpes zoster in her left back at an outside clinic. 20 mg/day of mirogabalin was also prescribed for her pain and she was not given any non-steroidal anti-inflammatory drugs. She had been treated as hypertension, and her medications were angiotensin receptor blocker (Olmesartan 20 mg/day) and calcium channel blocker (Azelnizipine 16 mg/day). Her recent serum creatinine level at regular checkup was 0.70 mg/dL and body mass index was 25.1 kg/m^2^. She did not experience any symptoms leading to dehydration, and was able to eat and drink as usual until the neurological symptoms developed.

**Fig. 1:**
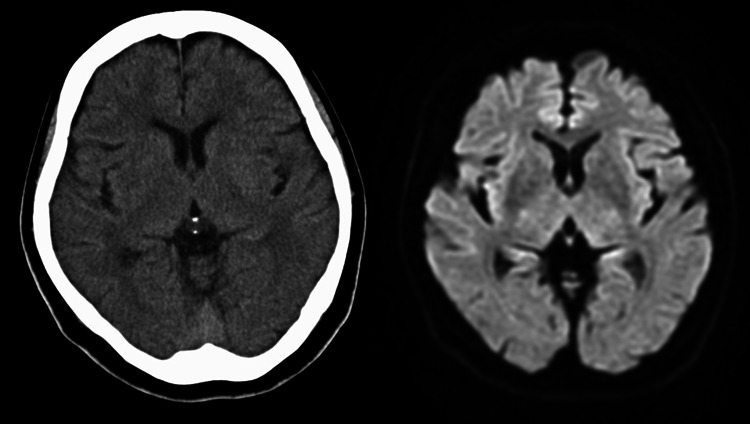
Head CT scan (left) at the ER showed no hemorrhagic lesions. MRI (right) showed neither fresh ischemic lesions nor abnormal signs of herpetic encephalitis

In two days, she visited an emergency room of a regional stroke care center with dysarthria and dexterity disorder and gait disturbance (Video1). Neither head CT nor MRI revealed intracranial lesions in her central nervous system (Fig. 1). Then, her serum creatinine levels turned out to be 4.63 mg/dL, therefore, she was transferred and admitted to our hospital on the following day. On admission, the patient was 146 cm, 57.5 kg. Vital signs are as follows: body temperature 36.3 ℃, blood pressure 117/74 mmHg, heart rate 89/minute, oxygen saturation in room air 95%, initial urinary volume was 20 ml/h. There were trace of several skin eruption in her left back. Blood and urine analyses on admission was shown in Table 1. The fractional excretion of sodium was 9.9%, which strongly indicated intrinsic AKI. Chest X-ray showed no congestive heart failure and abdominal CT showed no atrophy in her bilateral kidneys. In addition, as she did not present with fever, headache or neck stiffness, we denied viral encephalitis nor meningitis. Therefore, we assessed that she developed AKI due to acyclovir, then acyclovir-associated encephalopathy followed. She was afebrile, urinary and upper respiratory infection was denied. There were no obvious infectious symptoms other than herpes zoster.

**Table 1:** Laboratory data on admission

	Patient levels	Standard range
Blood analysis		
White blood cells	13,550/μL	3,300–8600/μL
Neutrophils	73.1%	42.4–75.0%
Eosinophils	4.6%	0.4–8.6%
Red blood cells	4.45 × 10^6^/μL	3.86–4.92 × 10^6^/μL
Hemoglobin	13.2 g/dL	11.6–14.8 g/dL
Hematocrit	39.4%	35.1–44.4%
Platelet	237 × 10^3^/μL	158–348 × 10^3^/μL
International normalized ratio	0.97	0.80–1.20
Active partial thromboplastin time	25.3 s	26.9–38.1 s
C-reactive protein	4.97 mg/dL	< 0.14 mg/dL
Total protein	6.8 g/dL	6.6–8.1 g/dL
Albumin	3.8 g/dL	4.1–5.1 g/dL
Total bilirubin	0.4 mg/dL	0.4–1.5 mg/dL
Aspartate aminotransferase	28U/L	13-30U/L
Alanine aminotransferase	24U/L	7-23U/L
Lactate dehydrogenase	261U/L	124-222U/L
Creatine phosphokinase	59U/L	41-153U/L
Blood urea nitrogen	51 mg/dL	8-20 mg/dL
Creatinine	4.63 mg/dL	0.46–0.79 mg/dL
Estimated glomerular filtration ratio	7.9 mL/min/1.73m^2^	
Sodium	141 mEq/L	138-145 mEq/L
Potassium	4.8 mEq/L	3.6–4.8 mEq/L
Glucose	126 mg/dL	73-109 mg/dL
Hemoglobin A1c	6.2%	4.9–6.0%
Ammonia	< 14 μg/dL	16-44 μg/dL
pH	7.310	7.350–7.450
Bicarbonate	22.8 mEq/L	22.0–26.0 mEq/L
Lactate	0.6 mM	< 2.0 mM
Urine analysis		
Specific gravity	1.006	1.005–1.030
Occult blood red cells	1–4/high power field	0–4/high power field
Protein	7 mg/dL	
Creatinine	32 mg/dL	
Protein/Creatinine	0.22	< 0.15
Sodium	70 mEq/L	
Fractional excretion of sodium	9.9%	
Potassium	8 mEq/L	
*N*-acetyl-β-D-glucosaminidase	2U/L	0-5U/L
β2-microglobuline	293 μg/dL	0-30 μg/dL

**Fig. 2:**
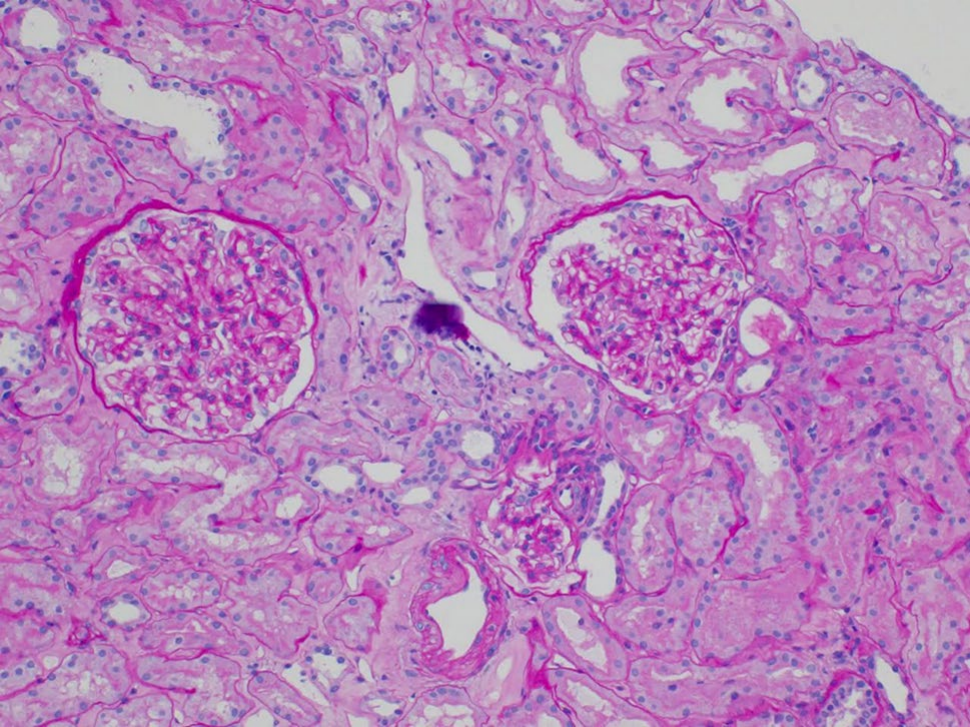
The renal biopsy revealed acute tubular injury with eosinophilic material in the tubular lumen (loss of epithelial cells), and some tubular epithelial cells with disappearing of nuclei

**Fig. 3:**
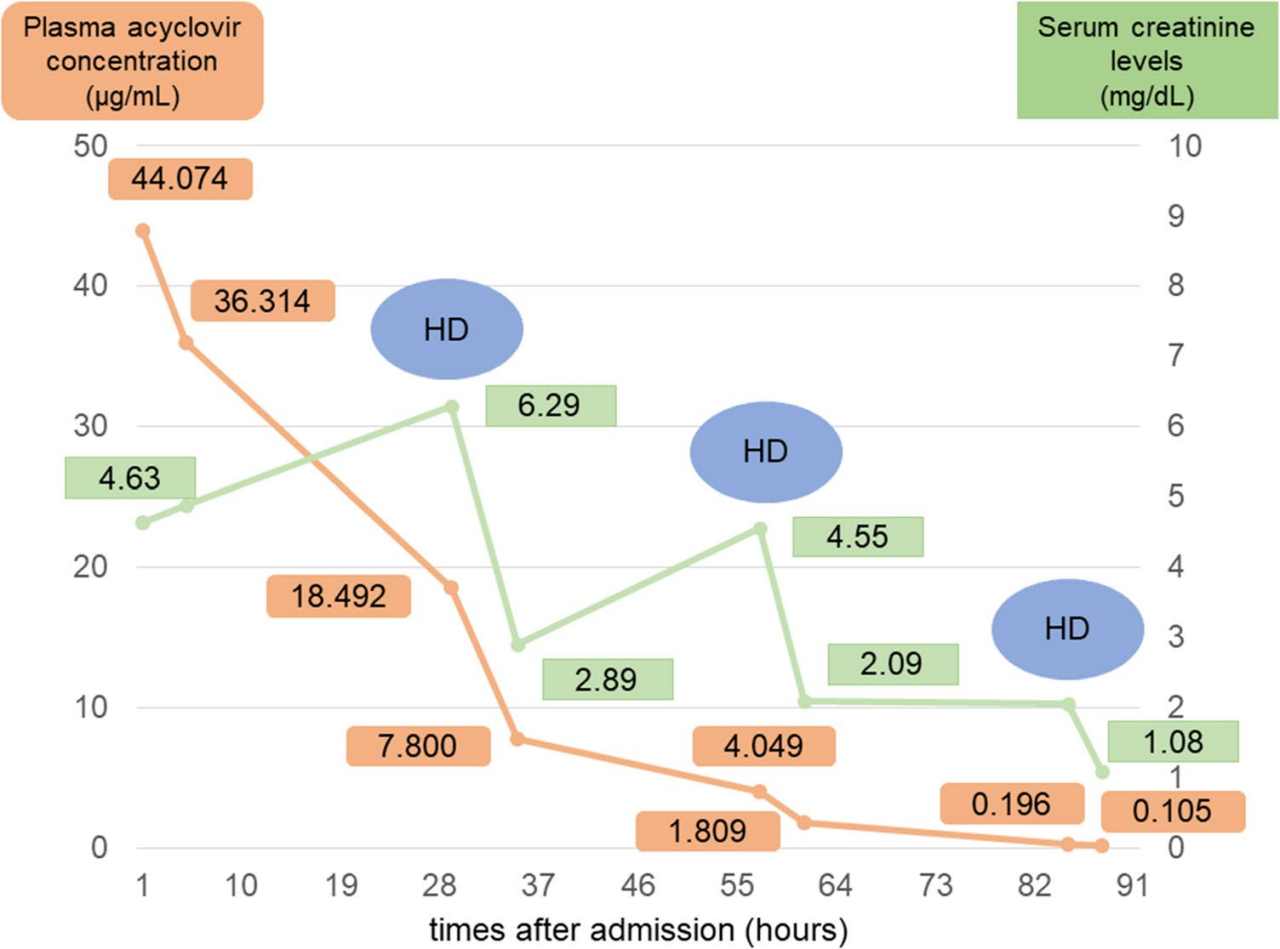
Her clinical course with serum creatinine levels and plasma acyclovir concentration. She needed total of three dialysis sessions

Hemodialysis was initiated on the second day of admission, we also performed renal biopsy on the same day. The renal biopsy revealed acute tubular injury with eosinophilic material in the tubular lumen (loss of epithelial cells), and some tubular epithelial cells with disappearing of nuclei, while neither glomerulonephritis nor interstitial nephritis were observed (Fig. 2). Both her serum creatinine levels and neurological symptom quickly improved by the 3 sessions of hemodialysis therapy. She was discharged on the 8th day of admission with almost normal renal function. Drug-induced lymphocyte stimulation test was not performed at this time. The serum creatinine level at the following outpatient clinic was 0.80 mg/dL, and she did not show any neurological sequela. Olmesartan was withdrawn during hospitalization, and restarted at this time. Afterward, the concentration of acyclovir in her serum at the time of admission revealed as 44 μg/mL, which is extremely high. Her clinical course with treatment was shown in Fig. 3. Acyclovir was quickly removed from serum by hemodialysis therapy. We finally diagnosed that AKI developed by the use of valacyclovir, then the delay of drug metabolism caused the increased serum concentration of acyclovir which leaded to encephalopathy.

## Discussion

Valacyclovir is widely administrated and generally well tolerated antiviral agent in the treatment of herpes simplex and varicella zoster virus infection, used by both oral and intravenously. However, the agent sometimes causes severe nephrotoxicity which results in AKI [2, 3]. Several mechanisms of this nephrotoxicity have been reported previously; one is renal tubular obstruction due to crystallization [5], the other is tubular dysfunction caused by a direct effect of acyclovir aldehyde [6], and a case of drug induced tubulointerstitial nephritis is also reported [2]. Valacyclovir is an oral prodrug of acyclovir. Valacyclovir has better oral bioavailability of 54.2% compared with acyclovir (10–20%) and 5 times longer plasma half time than acyclovir [1, 4]. Lam et al. reported that the hospital admission rate with AKI among the patients administrated oral acyclovir/valacyclovir was 0.27% [7]. Also, the significant risk factors for AKI are present in chronic kidney disease (4.02-fold of relative risk), loop diuretics use (1.85-fold), potassium-sparing diuretics use (1.68-fold), diabetes (1.51-fold), congestive heart failure (1.39-fold), angiotensin-converting enzyme inhibitors or angiotensin receptor blockers use (1.39-fold) and older age (1.07-fold) [7]. There is another report that obesity (BMI ≥ 30) is one of the risk factors of acyclovir-induced nephrotoxicity [8]. In this case, the findings of renal biopsy was relatively mild, compared with the level of serum Cr 4.63 mg/dl. Renal biopsy revealed hyalinosis in multiple small arteries, suggesting arteriosclerosis (not shown), her blood pressure on arrival was 117/74 mmHg, which was relatively low, therefore normotensive ischemic acute kidney injury is a possible explanation underlying in this case.

It is also sometimes reported that acyclovir-associated encephalopathy was caused by administration of both acyclovir and valacyclovir [4, 5]. Acyclovir encephalopathy is caused by an increased levels of serum 9-carboxymethoxymethylguanine, a metabolite of acyclovir [5]. When we diagnose acyclovir encephalopathy, we must keep in mind the possibility of concomitant herpetic encephalitis which has high mortality rate of up to 25% [6, 9]. In patients with herpetic encephalitis, we often observe fever, headache and meningitis-like neck stiffness. Also, head MRI shows some unspecific abnormal imaging [6, 9]. In our patient, although we did not perform her cerebrospinal fluid examination, we diagnosed her illness as acyclovir encephalopathy because she did not show any signs of meningitis not encephalitis. As for risk factors of acyclovir encephalopathy, Kenzaka et al. reported in their review of literatures that the 62.7% of the patients with acyclovir encephalopathy had severe renal insufficiency with hemodialysis or peritoneal dialysis therapy [5, 10, 11]. However, they also suggested that we have to pay attention even to the patients without renal insufficiency because acyclovir cause AKI regardless of the patients’ age and original renal function [5]. In our case, the patient was an elderly woman of relatively small stature, which could have caused relatively elevated blood levels and thereby renal damage. Then, it is assumed that the decrease in urinary excretion of acyclovir led to vicious cycle of further nephrotoxicity.

In the single-dose study of oral 1000 mg valacyclovir administration in healthy Japanese male volunteers, the maximum plasma acyclovir concentration and plasma half time were 5.84 ± 1.08 μg/mL and 3.55 ± 0.27 h, respectively [12]. Her serum concentration of 44 μg/mL on admission was considered extremely high level which is compatible with acyclovir encephalopathy. Also, the time course of acyclovir concentration presented in Fig. 3 indicated that the plasma half time before the first hemodialysis session was approximately 24 h, which is severely prolonged by AKI due to acyclovir nephrotoxicity. Once valacyclovir is orally administrated, it is converted in liver to its active form of acyclovir immediately [12]. The molecular weight of acyclovir is 225.20 and its protein binding rate is relatively low of 22–33% [13]. It is also reported that renal excretion of unchanged acyclovir and 9-carboxymethoxymethylguanine respectively reach to 43.1% and 5.0% in 24 h after oral administration of 1000 mg valacyclovir [12]. The renal function of the patient again plays an important role in the excretion of acyclovir. Thus, the renal replacement therapy, such as hemodialysis or continuous hemo-diafiltration, is effective for removing acyclovir when the patient had renal insufficiency, in the cases with AKI but also in those with chronic kidney disease. In our case, each hemodialysis session removed plasma acyclovir of 46–59% and improved the neurological symptoms promptly.

In conclusion, we must pay attention to the optimal dosage of acyclovir/valacyclovir prescription to the patients regardless of their age or renal function. When any neurological symptoms observed by acyclovir with concomitant sever renal insufficiency, it is suggested that immediate renal replacement therapy is recommended.

## Supplementary Information

Below is the link to the electronic supplementary material.

Supplementary file1 (MP4 11519 KB)

## References

[1] Weller S, Blum MR, Doucette M, Burnette T, Cederberg DM, de Miranda P (1993). Pharmacokinetics of the acyclovir prodrug valacyclovir after escalating single and multiple dose administration to normal volunteers. Clin Pharmacol Ther.

[2] Yildiz C, Ozsurekci Y, Gucer S, Cengiz AB, Topaloglu R (2013). Acute kidney injury due to acyclovir. CEN Case Rep.

[3] Chang YH, Tseng JY, Chen CY, Sung PL, Yeh CC, Yang MJ (2016). Acyclovir-induced nephrotoxicity in a pregnant woman with chickenpox. Taiwan J Obstet Gynecol.

[4] Kenzaka T, Sugimoto K, Goda K, Akita H (2021). Acute kidney injury and acyclovir-associated encephalopathy after administration of valacyclovir in an elderly person with normal kidney function. A case report and literature review. Medicine.

[5] Chowdhury MA, Derar N, Hasan S, Hinch B, Ratnam S, Assaly A (2016). Acyclovir-induced neurotoxicity: a case report and review of literature. Am J Ther.

[6] Kaewpoowat Q, Salazar L, Aguilera E, Wootton SH, Hasbun R (2016). Herpes simplex and varicella zoster CNS infections: clinical presentations, treatments and outcomes. Infection.

[7] Lam NN, Weir MA, Yao Z, Blake PG, Beyea MM, Gomes T (2013). Risk of acute kidney injury from oral acyclovir: A population-based study. Am J Kidney Dis.

[8] Barber KE, Wagner JL, Stover KR (2019). Impact of obesity on acyclovir-induced nephrotoxicity. Open Forum Infect Dis.

[9] Dworkin RH, Johnson RW, Breuer J, Gnann JW, Levin MJ, Backonja M (2007). Recommendations for the management of herpes zoster. Clin Infect Dis.

[10] Umoru GO, Shah PJ, Tariq F (2020). A case report of neurotoxicity after prolonged doses of acyclovir in a patient with renal dysfunction. J Pharm Pract.

[11] Sadjadi SA, Regmi S, Chau T (2018). Acyclovir neurotoxicity in a peritoneal dialysis patient: report of a case and review of the pharmacokinetics of acyclovir. Am J Case Rep.

[12] Azuma J, Aoki T, Yamamoto I, Seto Y, Tokuda K, Horiuchi M (1998). A phase I study of a new antiviral drug, valacyclovir hydrochloride (256U87), in healthy male volunteers. Rinsho Iyaku.

[13] Perry CM, Valacyclovir FD (1996). A review of its antiviral activity, pharmacokinetic properties and therapeutic efficacy in herpesvirus infections. Drugs.

